# Insulin augments mechanical strain-induced ERK activation and cyclooxygenase-2 expression in MG63 cells through integrins

**DOI:** 10.3892/etm.2013.1394

**Published:** 2013-11-08

**Authors:** XIAOHUAN ZHONG, HUIXIN WANG, XINCHUN JIAN

**Affiliations:** Department of Stomatology, Xiangya Hospital, Central South University, Changsha, Hunan 410008, P.R. China

**Keywords:** insulin, osteoblast, mechanical strain, cyclooxygenase-2, extracellular signal-regulated kinase

## Abstract

Insulin has been proposed to be a positive regulator of osteoblast proliferation and bone formation. *In vivo* mechanical loading is essential for maintaining skeletal integrity and bone mass. Since insulin and mechanical force activate similar signaling pathways in osteoblasts, it was hypothesized that insulin may affect mechanical stimulation in osteoblasts. The present study tested the hypothesis that insulin augments mechanical strain-induced signaling and early gene expression in MG63 cells via activation of the extracellular signal-regulated kinase (ERK) pathway and cyclooxygenase-2 (Cox-2) expression. Western blot analysis and quantitative polymerase chain reaction demonstrated respectively that insulin enhanced mechanical strain-induced ERK phosphorylation and Cox-2 expression levels in a dose-dependent manner. The effect of insulin on mechanical strain-induced Cox-2 expression was inhibited by blockade of the ERK pathway. In addition, echistatin, an inhibitor of integrin function, prevented the effects of insulin on mechanical strain-induced ERK phosphorylation and Cox-2 expression. The data obtained from this study suggested that insulin augments mechanical strain-induced Cox-2 expression levels via integrin-dependent activation of the ERK pathway in osteoblasts.

## Introduction

Mechanical loading is considered essential for maintaining skeletal integrity and bone mass. Previous studies have shown that mechanical stimulation regulates signaling, gene expression, proliferation and differentiation in osteoblasts (1–4). The application of mechanical loading to osteoblasts induces the activation of mitogen-activated protein kinases (MAPKs), such as extracellular signal-regulated kinase (ERK), c-jun-NH_2_-terminal kinase (JNK) and p38 MAPK, and expression of numerous osteogenic genes, including cyclooxygenase-2 (Cox-2), early growth response-1 (Egr-1) and transcription factor c-fos (5,6).

Type 1 diabetes mellitus, which is characterized by a lack of insulin production, is associated with decreased skeletal mass (7–9). Studies have shown that insulin is important in the regulation of bone metabolism (10,11). Insulin is capable of promoting osteoblast proliferation and differentiation, collagen synthesis and alkaline phosphatase production (12–14). Insulin deficiency accelerates bone loss and is potentially the main cause of osteoporosis in patients with type 1 diabetes mellitus (15). Following the binding of insulin to its receptor, which is present on osteoblasts (10,11,13), downstream signaling cascades, including the phosphatidylinositol 3-kinase (PI3K) and MAPK pathways, are activated (16). It has been shown that insulin stimulates osteoblast proliferation and differentiation through the ERK and PI3K pathways (13), which are also involved in osteoblast response to mechanical stimulation (5,17). Although insulin and mechanical forces activate similar signaling pathways in osteoblasts, whether there is convergence of these activated signaling pathways has yet to be elucidated.

Integrins, the main receptors that connect the cytoskeleton and extracellular matrix (ECM), have been shown to have key roles in linking ECM components with various intracellular signaling mechanisms (18), including the transmission of mechanical stresses into chemical signals in a wide variety of cells seeded on the ECM (19). Human osteoblasts express several types of integrins. Integrins mediate the expression of bone formation-associated genes in osteoblasts in response to mechanical stimulation via the ERK, JNK and p38 MAPK pathways (5). A previous study demonstrated the crucial role of integrin β1 signaling events in mediating cross-talk of insulin action (20). Although integrins have been recognized as mechanosensors in osteoblasts in response to mechanical stimuli and regulators of insulin signaling, whether insulin regulates the mechanical responsiveness of signaling and gene expression in osteoblasts through integrins has yet to be elucidated.

The aim of the present study was to investigate the role of insulin in regulating the response of osteoblasts to mechanical stimulation by examining changes in the activation of the ERK pathway and the expression of Cox-2.

## Materials and methods

### Materials

MG63 cells were obtained from the American Type Culture Collection (Manassas, VA, USA). Fetal bovine serum (FBS) was obtained from HyClone (Logan, UT, USA) and Dulbecco's modified Eagle medium (DMEM) was purchased from Gibco-BRL (Grand Island, NY, USA). Mouse monoclonal antibodies against ERK2 (sc-1647) and phospho-ERK1/2 (sc-7383), and the ERK inhibitor, PD98059, were obtained from Santa Cruz Biotechnology, Inc. (Santa Cruz, CA, USA). Insulin and echistatin were purchased from Sigma (St. Louis, MO, USA). A First Strand cDNA Synthesis kit was obtained from Fermentas (Fermentas UAB, Vilnius, Lithuania) and a LightCycler^®^ 480 SYBR Green I Master was purchased from Roche Applied Science (Indianapolis, IN, USA). All additional chemicals of reagent grade were obtained from Sigma unless otherwise noted. The study protocol conforms to the ethical guidelines of the World Medical Association, Declaration of Helsinki. The experimental protocols were approved by the Ethics Committee of Xiangya Hospital.

### Cell culture

Cells were cultured in DMEM supplemented with 10% FBS and 1% penicillin/streptomycin at 37°C in a humidified atmosphere of 95% air and 5% CO_2_. Upon reaching confluence, cells were trypsinized and seeded into the force-loading plate at a density of 1×10^4^ cells/cm^2^. The force-loading plates were made using the method described previously (3). The cells were incubated in DMEM supplemented with 10% FBS (pretreated with charcoal to remove endogenous insulin in the serum) and 1% penicillin/streptomycin for 48 h. The medium was then changed to FBS-free medium for 24 h to equalize cell growth prior to the experiments.

### Experimental groups

The cells were divided into three groups. Group 1: the cells were maintained as a control or pretreated with varied doses of insulin (0, 10 and 100 nm) for 4 h, then exposed to tensile stress for 10 min. Group 2A: the cells were maintained as a control or pretreated with varied doses of insulin (0, 10 and 100 nm) for 4 h, then exposed to tensile stress for 1 h. Group 2B: the cells were pretreated with DMSO or PD98059 for 1 h, stimulated with insulin (10 nm) for 4 h, then followed by exposure to tensile stress for 10 min. Group 3: the cells were maintained as a control or pretreated with echistatin for 24 h, stimulated with insulin (10 nm) for 4 h, then exposed to tensile stress for 10 min or 1 h.

### Mechanical stress application

The plates were subjected to cyclic uniaxial tensile strain by the four-point bending system at a frequency of 0.5 Hz, and cells were loaded with tensile stress at 2,000 μ strain, using a method that has been previously characterized and described in detail (3). In the group without the mechanical stimulus, cells were seeded into similar plates and incubated in the same incubator without mechanical stress loading. For certain experiments, MG63 cells were incubated with 30 μM PD98059 (the specific inhibitor for ERK) for 1 h and 50 nM echistatin (integrin antagonist) for 24 h prior to stimulation with insulin or exposure to mechanical stress. The treatments were repeated three times.

### RNA isolation and quantitative polymerase chain reaction (qPCR)

Following the harvesting of the cells, total RNA was extracted using the guanidium isothiocyanate/phenochloroform method. The samples were lysed in 1 ml TRI reagent. The homogenate was stored at room temperature for 5 min to complete the dissociation of nucleoprotein complexes, at which point 0.2 ml chloroform was added to the homogenate, followed by centrifugation at 12,000 × g for 15 min. After centrifugation, RNA was precipitated from the upper aqueous phase by adding 0.5 ml isopropanol to the tubes and then centrifuging at 12,000 × g for 10 min. After this centrifugation step, the RNA pellet was washed with 75% ethanol and centrifuged at 7,000 × g for 5 min. The RNA pellet was air dried and dissolved in 75 μl H_2_O at 60°C for 15 min. Total RNA was quantified using spectrophotometric analysis of the absorbance at 260 nm (ultraviolet spectrophotometer UV-752; Shanghai optical instrument factory, Shanghai, China). RNA was reverse-transcribed using the First Strand cDNA Synthesis kit (Fermentas) in accordance with the manufacturer's instruction. cDNA was amplified by qPCR using the LightCycler instrument (Roche Applied Science). Sequences of all PCR primers are shown in Table I. Data were analyzed using the 2^−ΔΔCT^ method by Livak and Schmittgen (21), using the housekeeping gene β-actin to calculate the ΔCT. The control was used at each time point to calculate the −ΔΔCT.

### Western blot analysis

Following mechanical stress loading, the treated cells and corresponding controls were immediately washed twice with ice-cold phosphate-buffered saline (PBS) and lysed with buffer containing 1% NP-40, 0.5% sodium deoxycholate, 0.1% SDS and a protease inhibitor mixture (phenylmethylsulfonyl fluoride, aprotinin and sodium orthovanadate). Protein concentration was measured with the bicinchoninic acid (BCA) protein assay. Briefly, 2 μg protein in 500 μl was prepared, then 500 μl working solution was added to each 500 μl sample and mixed. The samples were incubated for 60 min at 60°C, cooled and analyzed at 562 nm. Protein extracts (10 μg) were separated on 10% SDS-polyacrylamide gels and transblotted to polyvinylidene difluoride membranes for western blot analysis. The target bands were determined according to the molecular weight of the target proteins [phosphorylated ERK1 (p-ERK1), p-ERK2 and ERK2, weighing 44, 42 and 42 kDa, respectively]. The band intensity ratio of p-ERK1/2 and ERK2 (p-ERK/ERK) was analyzed to assess ERK1/2 activation (normalized to β-actin).

### Statistical analyses

Results are shown as the mean ± standard deviation. Statistical analysis was performed using an independent Student's t-test for two groups of data and analysis of variance followed by Scheffe's test for multiple comparisons. P<0.05 was considered to indicate a statistically significant difference.

## Results

### Insulin augments tensile stress-induced ERK phosphorylation in a dose-dependent manner in MG63 cells

**Figure 1:**
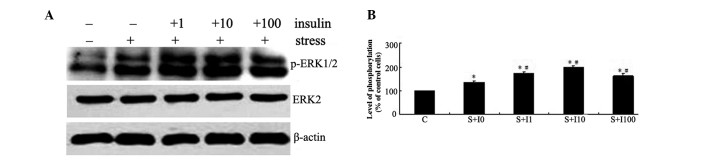
Insulin augments tensile stress-induced ERK phosphorylation in MG63 cells. MG63 cells were kept as controls or pretreated with varied doses of insulin (0, 1, 10 and 100 nM) for 4 h and then exposed to tensile stress for 10 min. ERK phosphorylation was determined using western blot analysis. The results are expressed as the mean ± standard deviation. ^*^P<0.05 vs. unstimulated control cells; ^#^P<0.05 vs. stressed cells without insulin pretreatment. ERK, extracellular signal-regulated kinase; C, control; I, insulin; S, tensile stress.

The initial experiment examined the effects of insulin on mechanical strain-induced ERK phosphorylation. The phosphorylation of ERK was determined by western blot analysis (Fig. 1A). MG63 cells were pretreated with varied doses of insulin (0, 1, 10 and 100 nM) for 4 h and then exposed to tensile stress for 10 min. The activation of ERK in these cells was compared with that in the cells not pretreated with insulin or exposed to tensile stress. As shown in Fig. 1, mechanical strain resulted in increased ERK phosphorylation following tensile stress stimulation in MG63 cells (P<0.05). Pretreatment of MG63 cells with insulin prior to exposure to tensile stress resulted in a dose-dependent increase in ERK phosphorylation in these cells compared with the cells exposed to tensile stress alone (P<0.05). The highest levels of ERK phosphorylation were observed in the group pretreated with 10 nM insulin (P<0.05; Fig. 1B).

### Insulin augments tensile stress-induced Cox-2 expression levels via the ERK pathway in MG63 cells

**Figure 2:**
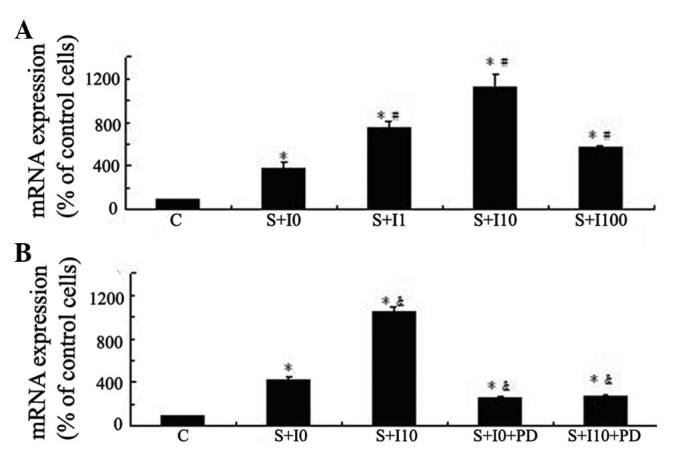
Insulin augments tensile stress-induced Cox-2 expression levels via the ERK pathway in MG63 cells. (A) MG63 cells were kept as controls or pretreated with varied doses of insulin (0, 1, 10 and 100 nM) for 4 h and then exposed to tensile stress for 1 h. (B) MG63 cells were pretreated with vehicle control DMSO or PD98059 (30 μM) for 1 h then stimulated with insulin (10 nM) for 4 h, followed by exposure to tensile stress for 1 h. Cox-2 expression levels were determined using qPCR. The results are expressed as the mean ± standard deviation. ^*^P<0.05 vs. unstimulated control cells; ^#^P<0.05 vs. stressed cells without insulin pretreatment; ^&^P<0.05 vs. stressed cells without insulin and PD pretreatment. Cox-2, cyclooxygenase-2; ERK, extracellular signal-regulated kinase; DMSO, dimethylsulfoxide; qPCR, quantitative polymerase chain reaction; C, control; I, insulin; S, tensile stress; PD, PD98059.

To investigate the enhancing effect of insulin on mechanical strain-induced Cox-2 expression levels, MG63 cells were treated with varied doses of insulin (0, 1, 10 and 100 nM) for 4 h and then exposed to tensile stress for 1 h. Cox-2 mRNA expression levels in these cells was measured using qPCR and compared with those in the cells not pretreated with insulin or exposed to tensile stress. Mechanical strain resulted in increased Cox-2 mRNA expression levels in MG63 cells. Insulin augmented mechanical strain-induced Cox-2 mRNA expression in a dose-dependent manner and the most notable effect was observed in the group pretreated with 10 nM insulin (P<0.05; Fig. 2A). Pretreatment of MG63 cells with PD98059 inhibited the insulin-augmented mechanical strain-induced Cox-2 expression levels, suggesting that the effect of insulin on tensile stress-induced Cox-2 expression levels in MG63 cells was mediated by the ERK pathway (P<0.05; Fig. 2B).

### Insulin augmentation of tensile stress-induced ERK phosphorylation and Cox-2 expression levels in MG63 cells is mediated by integrins

**Figure 3:**
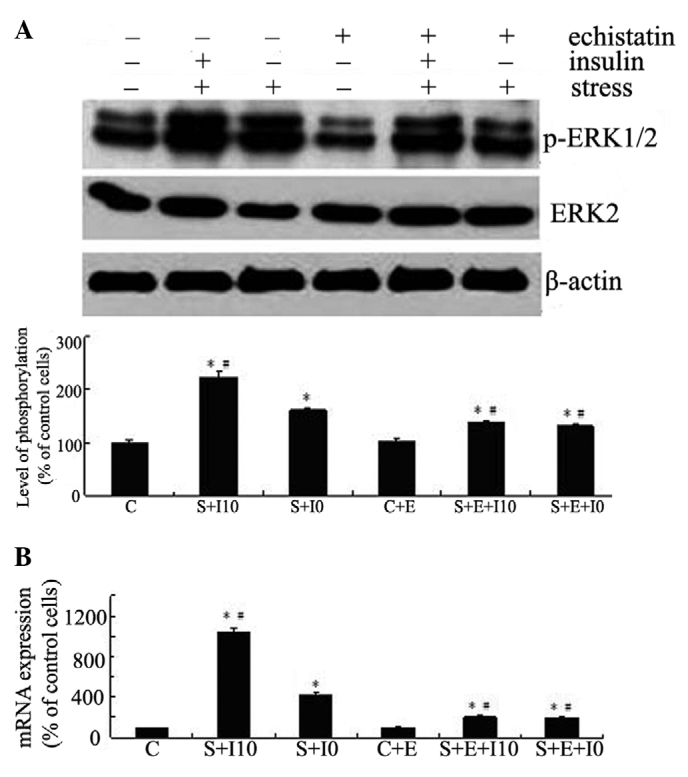
Insulin augmentation of tensile stress-induced ERK phosphorylation and Cox-2 expression levels in MG63 cells is mediated by integrins. MG63 cells were kept as controls or pretreated with echistatin (50 nM) for 24 h, stimulated with insulin (10 nM) for 4 h and exposed to tensile stress for 10 min (for ERK) or 1 h (for Cox-2). (A) ERK phosphorylation was determined using western blot analysis. (B) Cox-2 expression levels were determined using qPCR. The results are expressed as the mean ± standard deviation. ^*^P<0.05 vs. unstimulated control cells; ^#^P<0.05 vs. stressed cells without echistatin and insulin pretreatment. Cox-2, cyclooxygenase-2; ERK, extracellular signal-regulated kinase; qPCR, quantitative polymerase chain reaction; C, control; E, echistatin; I, insulin; S, tensile stress.

MG63 cells were kept as controls or pretreated with echistatin for 24 h, stimulated with insulin for 4 h, and exposed to tensile stress for 10 min (for ERK) or 1 h (for Cox-2). The ERK phosphorylation and Cox-2 expression levels were examined by western blotting and qPCR, respectively. As shown in Fig. 3, suppression of integrin function inhibited not only the tensile stress-induced ERK phosphorylation (Fig. 3A) and Cox-2 expression (Fig. 3B), but also the effects of insulin on the tensile stress-induced activation of this signaling pathway and expression of this gene (P<0.05).

## Discussion

Osteoblasts may exhibit distinct responses to mechanical loading under various *in vivo* conditions, as defective cellular responses to mechanical loading have been observed in patients with musculoskeletal diseases, including disuse osteoporosis, senile osteoporosis and osteoarthritis (22,23). The concentration of insulin is much lower in patients with type 1 diabetes mellitus than in healthy individuals, which may impact the skeletal response to mechanical loading in these patients. Therefore, this investigation explored the potential effects of insulin on the response of osteoblasts to mechanical stimulation by examining changes in the activation of the ERK pathway and the expression of Cox-2.

ERK is regarded as a key point of upstream signal transduction pathway in the cellular response to extracellular signals (24), including mechanical signals (1,5). ERK is rapidly activated by mechanical stimuli (5). It is involved in the increased expression of bone formation-associated genes (Egr-1, c-fos and Cox-2) in osteoblasts induced by mechanical stimulation (5), and in osteoblast proliferation and differentiation induced by insulin (13). In the present study we demonstrated for the first time, to the best of our knowledge, that insulin augments tensile stress-induced ERK phosphorylation in a dose-dependent manner in MG63 cells. The results indicated that insulin upregulates the mechanosensitivity of osteoblasts via the ERK pathway, which suggests that the mechanical sensitivity of bone may be reduced in patients with type 1 diabetes mellitus. Variations in insulin concentration may also affect the mechanosensitivity of osteoblasts.

Cox-2 is a rate-limiting enzyme in the regulation of prostaglandin (PG) synthesis in bone with Cox-1. This enzyme appears to be important for the osteogenic response of bone to exogenous mechanical loading. Cox-2 is immediately upregulated in response to mechanical stimulation in osteoblasts (25), and has been shown to be important in bone formation *in vivo*(26). Inhibition of Cox-2 expression significantly decreased bone formation rates induced by mechanical stimulation in rats (26). Through a series of systematic studies, we demonstrated for the first time that insulin augments tensile stress-induced Cox-2 expression levels in a dose-dependent manner in MG63 cells. Furthermore, the increases in Cox-2 expression levels were inhibited by blockade of the ERK pathway. These results indicate that insulin modulates the tensile stress-induced Cox-2 expression levels in osteoblasts through the ERK pathway, which suggests that insulin may affect the signaling and function of bone cells in response to mechanical forces and, consequently, affect the mechanoresponsiveness of bone in the process of bone formation. Insulin deficiency may decrease Cox-2 expression levels in patients with type 1 diabetes mellitus and subsequently decrease bone formation.

A previous study demonstrated that adhesive ability and integrin-mediated signaling activation were lower in osteoblasts derived from patients with osteoporosis than those from healthy individuals (27), which indicates that integrins may be involved in the development of osteoporosis in patients with type 1 diabetes mellitus. Previous studies have also suggested an important role of integrins in regulating insulin signaling (28). For example, engagement of the β1 subunit containing integrin receptors was observed to increase insulin-stimulated insulin receptor substrate (IRS) phosphorylation and activate downstream signaling cascades, such as IRS-associated PI3K and protein kinase B/Akt (28). In addition, previous studies have shown that insulin and insulin-like growth factor (IGF)-I are capable of binding to each other's receptors, and both receptors phosphorylate IRS proteins on the same tyrosine residues to recruit and activate downstream signaling cascades, such as PI3K and MAPK pathways (10,11,16,29). Furthermore, IGF-I regulates the mechanical responsiveness of signaling and proliferation in osteoblasts via integrins (30). Consequently, it was hypothesized that insulin and IGF-I may share a common signaling pathway when they act on cells, and integrins may be partly involved in insulin regulation of mechanical responsiveness in osteoblasts. The results of the present study showed that suppression of integrin functions inhibited the enhancing effects of insulin on mechanical strain-induced ERK phosphorylation and Cox-2 expression. These results suggested that integrins are important in the insulin-modulated responsiveness of signaling and gene expression in osteoblasts in response to mechanical stimulation, which consequently may influence the formation and remodeling of bone.

In conclusion, this study demonstrated for the first time, to the best of our knowledge, that insulin upregulates mechanical strain-induced ERK signaling and Cox-2 expression in MG63 cells. The effect of insulin on signaling and gene expression is mediated by integrins. These findings show that insulin may influence the mechanical response of osteoblasts and provide mechanistic insights into the cross-talk between the integrin and insulin signaling pathways. In addition, the results of this study may aid the future development of pharmacological therapies for type 1 diabetes mellitus. Further validation of these results requires studies focusing on normal, primary osteoblasts.

## Figures and Tables

**Table I:** Primer sequences used for qPCR.

Gene name	Primer sequence	Size (bp)	Annealing Temperature (°C)
Cox-2	TCACGCATCAGTTTTTCAAGATC ACCGTAAATATGATTTAAGTCCAC	94	60
β-actin	AAATCGTCCGTGACATCAAG GGAAGGAAGGCTGG AAGA GA	180	60

qPCR, quantitative polymerase chain reaction; Cox-2, cylooxygenase-2.
